# Quality of care in pediatrics: the organization of a children’s hospital

**DOI:** 10.1186/1824-7288-40-S1-A37

**Published:** 2014-08-11

**Authors:** Massimiliano Raponi

**Affiliations:** 1 Medical Director of Bambino Gesù Children’s Hospital, Rome, 00165, Italy

## 

The Bambino Gesù Children's Hospital (OPBG) is a Research and Health Care Institute (IRCCS), organized in four sites located in Lazio Region (Roma Gianicolo, Roma S. Paolo, Palidoro, S. Marinella) and three sites located in Calabria, Basilicata and Sicily respectively. The OPBG as IRCCS, must always continue and reinforce its commitment in the integration of research and clinical practice, and transfer into the clinical practices of the knowledge produced by scientific research.

The OPBG Medical Direction is organized according to a medical and nursing multidisciplinary model and involves the interaction of a variety of professionals who work for the achievement of organizational and healthcare goals. This organization has allowed the implementation of multiple activities aimed to fully empower the staff, promote professionalism, develop awareness in relation to the needs of patients and their families and increase the focus on the continuous improvement of quality of care.

The IRCCS Bambino Gesù Children’s Hospital has long since launched a series of initiatives aimed at the continuous improving in quality of care, organized according to a model of integrated clinical governance, which focuses on the whole patient and the care plan family. This approach integrates the treatment options with the patient/family’s expectations in a model that imagine doctor and patient/family as cooperating partners (Family - Centered Care). In this context it is also ensured the empowerment of the patient and his family, who become a central point in decision-making and treatment processes.

Operationally, the Hospital every year prepare a Corporate Program for Continuous Improvement of Healthcare Quality, which identifies the objectives, even in terms of quantities, to be achieved in the year to ensure the continuous improvement of quality of care and monitors the achievement of targets set for the previous year.

The initiatives that are most representative of continuous promotion of healthcare quality carried out in the Hospital include: surveillance , control and prevention of adverse events; evaluating of the clinical and organizational appropriateness; production , dissemination and implementation of clinical protocols and evidence-based clinical pathway; surveillance , control and prevention of healthcare associated infections; design of clinical records that favor appropriateness and patient safety.

